# An Active Learning Classifier for Further Reducing Diabetic Retinopathy Screening System Cost

**DOI:** 10.1155/2016/4345936

**Published:** 2016-08-29

**Authors:** Yinan Zhang, Mingqiang An

**Affiliations:** ^1^School of Computer Science and Technology, Beijing Institute of Technology, Beijing 100081, China; ^2^College of Computer Science and Information Engineering, Tianjin University of Science and Technology, Tianjin 300222, China; ^3^College of Science, Tianjin University of Science and Technology, Tianjin 300222, China

## Abstract

Diabetic retinopathy (DR) screening system raises a financial problem. For further reducing DR screening cost, an active learning classifier is proposed in this paper. Our approach identifies retinal images based on features extracted by anatomical part recognition and lesion detection algorithms. Kernel extreme learning machine (KELM) is a rapid classifier for solving classification problems in high dimensional space. Both active learning and ensemble technique elevate performance of KELM when using small training dataset. The committee only proposes necessary manual work to doctor for saving cost. On the publicly available Messidor database, our classifier is trained with 20%–35% of labeled retinal images and comparative classifiers are trained with 80% of labeled retinal images. Results show that our classifier can achieve better classification accuracy than Classification and Regression Tree, radial basis function SVM, Multilayer Perceptron SVM, Linear SVM, and *K* Nearest Neighbor. Empirical experiments suggest that our active learning classifier is efficient for further reducing DR screening cost.

## 1. Introduction

Diabetic retinopathy (DR) [1] is one of the most common causes of blindness in diabetic mellitus research [2]. Millions of diabetic patients suffer from DR. DR not only deprives patients' sight [3] but also brings heavy burden to their family and society [4]. In 2012 [5], 29.1 million Americans (9.3% of the population) were diagnosed with diabetes. A more serious problem is that 76% of those patients were becoming with worsening diabetes. Each year, approximately 1.4 million Americans are diagnosed with diabetes. With the development of diabetes, about 40% of patients may lose sight from DR [6]. Recently, new technique named optical coherence tomography (OCT) is popular in developed countries. OCT can perform cross-sectional imaging, but OCT is still too expensive for many areas which are economically underdeveloped. Thus DR screening system is still useful for diabetic patients in many low income areas. This challenging problem causes a demand of a better computer-aided DR screening system [7, 8].

Many computer-aided screening systems can reduce massive manual screening effectively [9, 10]. Gardner et al. [11] propose an automatic DR screening system with artificial neural network. Most of computer-aided DR screening researches focus on reducing and improving doctor's work. It is noteworthy that Liew et al. [12] point out a critical issue; this issue is about accuracy and cost effectiveness. A typical DR screening hardware system includes but is not limited to high resolution camera, computing system, and storage system. The software system for DR screening system mainly contains three major parts: image processing [13], feature extraction [14], and classification [15] (automatic diagnosis result of computer). The architecture of computer-aided DR screening hardware system is clear and stable nowadays, but software system still has much space for development. Classification is an important breakthrough for improving DR screening system, especially when applying active learning method rather than supervised learning or unsupervised learning method.

However, to build an automatic computer-aided screening system raised a financial problem [16]. A DR screening system faces three major requirements nowadays. First, when a company builds a DR screening system for medical purpose, the accuracy is a key measurement. Second, hospital administrators need that this DR system not only can make classification automatically but also can save more money and time when it is running in the future. Third, the DR screening system should raise meaningful queries to doctors as many as possible, and cases that can be easily diagnosed by computer should be queried as little as possible. Therefore, a DR screening system should further have the following three characters: (1) more accuracy, (2) smaller training dataset, and (3) active learning.

For solving the above problems, we propose an ensemble-kernel extreme learning machine (KELM) based active learning with querying by committee classifier. Below are the major contributions/conclusions of our work:

1. Retinal image is easy to snap, but manually diagnosing a result is of high cost.
2. Kernel technique is suitable for classifying retinal images which is related to classification in high dimensional spaces.
3. Ensemble learning (bagging technique) can elevate classifier's performance. Particularly, overfitting occurs when training set is small.
4. Active learning can further reduce the size of training dataset compared to traditional machine learning method in DR screening system.
5. The committee can avoid unnecessary queries to doctor; this is distinctive to other state-of-the-art DR screening systems.

This paper is organized as follows: Section 2 shows background of retinal images and related works, Section 3 presents the details of the proposed classifier, and Section 4 presents empirical experiment and results. Conclusions are drawn in the final section.

## 2. Retinal Images and Related Works

### 2.1. Retinal Image and Detections

**Figure 1:**
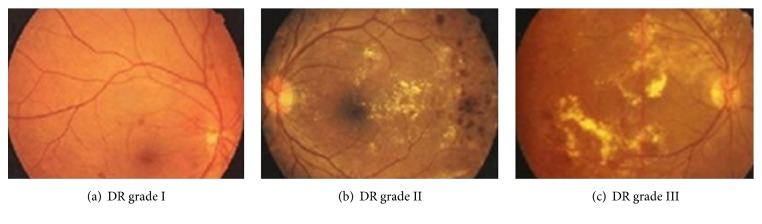
Representative images having different grades (I, II, and III).

**Figure 2:**
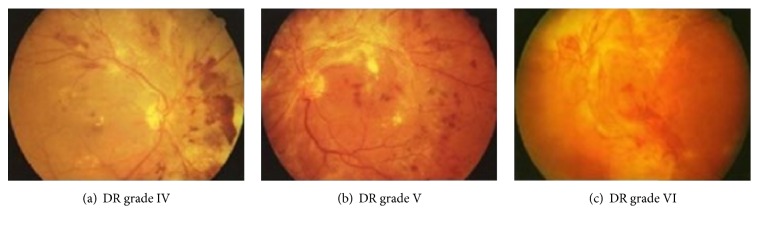
Representative images having different grades (IV, V, and VI).

Figure 1 shows DR grade [17]: I, II, and III.  Figure 2 shows DR grade: IV, V, and VI. Microaneurysm appears as tiny red dots in Figure 1; with the worsening of diabetes, exudates occur as primary signs of diabetic retinopathy. In Figures 1 and 2, inhomogeneity appears and it can lead to loss of sight.

Doctors give diagnosis results based on 3 major lesions: microaneurysm, exudates, and inhomogeneity. Moreover, there are two useful anatomical detections: macula and optic disc. In Table 1, five essential detections of DR screening are listed.

### 2.2. Classic DR Screening System Architecture

**Figure 3:**
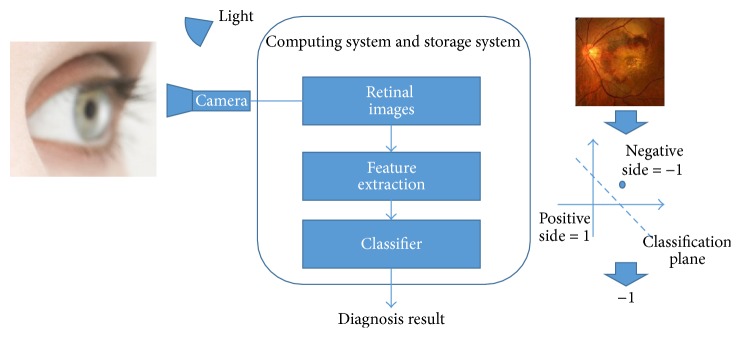
DR screening system architecture.

DR screening system [19] captures retinal images and gives diagnosis results. The classic architecture of DR screening system is shown in Figure 3.

A high resolution camera is used for capturing retinal images. Then, retinal images are saved into storage system. Usually, there is a preprocessing for retinal images; this process enhances image contrast and so forth. In the next step, multiple features of retinal images are extracted by image algorithms. Extracted features are represented in high dimensional space. Therefore original retinal image is mapped into high dimensional space. One retinal image is presented as a vector (or a dot) in this high dimensional space. Finally, a trained classifier gives a binary result (−1/1 or 1/0). This binary result indicates that the vector belongs to the “positive” side or the “negative” side. Figure 3 also exemplifies a brief workflow in two-dimensional space.

Many DR screening system studies focus on the performance of accuracy measurement. Dabramoff et al. [20] pointed out that DR screening system is an investigated field. Fleming et al. [21] showed that reducing mass manual effort is the key of creating DR screening system. Meanwhile, several researchers focus on automatic diagnosis of patients having DR [22]. Even though those researches and applications save massive manual work, DR screening system cost can be further reduced.

## 3. Ensemble Extreme Learning Machine Based Active Learning Classifier with Query by Committee

In this section, the proposed classifier is described in detail. With the consideration of accuracy, time consuming, computing resource consuming, high dimensional features classification, and reducing artificial labeling, we adapt kernel extreme learning machine (KELM) and then we use ensemble learning (bagging technique) to solve overfitting problem. Moreover, the bagging-KELM can be trained in parallel computing architecture.

### 3.1. Active Learning with Query by Committee

Active learning [23] has control over instances, once active learning reaches query paradigm, in which the committee can assign new artificial labeling task for human. Query by committee (QBC) [24] is a learning method which adopts decision of a committee to decide an unlabeled instance should be asked for artificial labeling or not. Once an artificial labeling task is finished, the new artificial labeled instance is added into training set. Therefore, the committee reduces testing instances and enlarges training set with asking for artificial labeling work. Since QBC has control over instances from which it learns, QBC maintains a group of hypotheses from training set; those hypotheses represent the version space. For real word problems, the size of committee should be big enough.

**Figure 4:**
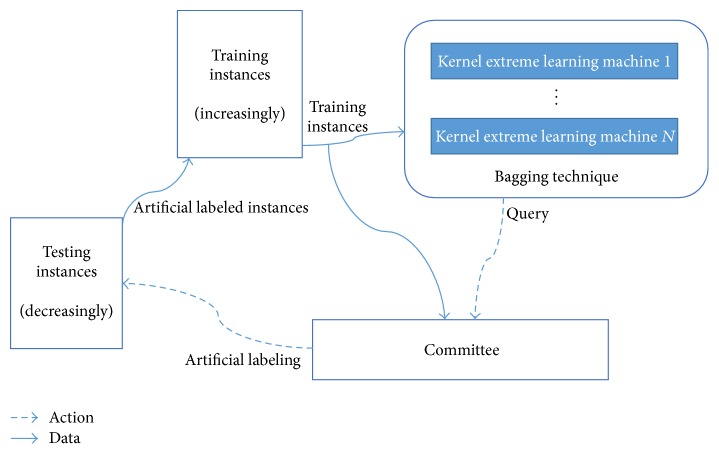
Active learning with query with committee.

Figure 4 shows the proposed method. Our approach contains 3 cyclic steps.

**Step 1 .**

KELM with bagging technique and committee are trained synchronously. Initial training instances consist of extracted features from DR images and corresponding artificial label marks.

**Step 2 .**

After the training procedure, the committee can propose necessary queries for bagging-KELM.

**Step 3 .**

In the testing procedure, both bagging-KELM and the committee receive testing instances and then bagging-KELM asks permission from the committee. If committee agrees with bagging-KELM, bagging-KELM gives a hypothesis for an unlabeled instance as final diagnosis result. However, if committee gives disagreement, the committee proposes the unlabeled retinal image to human doctor (this increases training instances).

To conclude, our approach is dealing with 3 optimization problems: (1) increasing training dataset as little as possible, (2) increasing training dataset with necessary queries, and (3) decreasing testing dataset with control.

### 3.2. Kernel Extreme Learning Machine

Extreme learning machine (ELM) [25] is a fast and accurate single-forward layer feedforward neural network classification algorithm proposed by Huang et al. Different from traditional neural networks, ELM assigns perceptron with random weights in the input layer and then the weights of output layer can be calculated catalytically by finding the least square solution. Therefore, ELM is faster than other learning algorithms for neural network; the time cost is extremely low.

For diabetic retinopathy screening, given a training dataset *X* with *N* labeled instances (*x*_*i*_, *t*_*i*_), *i* = 1,2,…, *N*, where each *x*_*i*_ is an *n* dimensional vector, *x*_*i*_ = [*x* ^1^, *x* ^2^, *x* ^3^,…,*x* ^*n*^]^*T*^ ∈ *R* ^*n*^, and *t*_*i*_ is an indicating label of corresponding instance, the output of signal-layer forward network with *M* perceptrons in middle layer can be calculated as follows:

(1) ∑i=1Mβigixj=∑i=1Mβigwi+xj+bi=tj,j=1,2,…,N,

where *w*_*i*_ is the weights connecting the *i*th middle perceptron with the input perceptron. *β*_*i*_ is the weights connecting the *i*th hidden perceptron with the output perceptron, and *b*_*i*_ is the bias of the *i*th hidden perceptron.

*g*(·) denotes nonlinear activation function; some classical activation functions are listed as follows:

(1) Sigmoid function:
  (2) $$\begin{array}{c} G\left(\;a,b,x\;\right)=\frac{1}{1+\exp\left(-\left(a\cdot x+b\right)\right)}. \end{array}$$
(2) Fourier function:
  (3) $$\begin{array}{c} G\left(\;a,b,x\;\right)=\sin\left(\;a\cdot x+b\;\right). \end{array}$$
(3) Hard limit function:
  (4) $$\begin{array}{c} G\left(\;a,b,x\;\right)=\left\{\begin{array}{cc} 1, & if\;\;a\cdot x-b\geq 0\\ 0, & otherwise. \end{array}\right. \end{array}$$
(4) Gaussian function:
  (5) $$\begin{array}{c} G\left(\;a,b,x\;\right)=\exp\left(\;-b{\left\|\;x-a\;\right\|}^{2}\;\right). \end{array}$$
(5) Multiquadrics function:
  (6) $$\begin{array}{c} G\left(\;a,b,x\;\right)={\left(\;{\left\|\;x-a\;\right\|}^{2}+b^{2}\;\right)}^{1/2}. \end{array}$$

Equation (1) can be expressed in a compact equation as follows:

(7) $$\begin{array}{c} H\beta =T, \end{array}$$

where *H* is the middle layer output matrix:

(8) $$\begin{array}{c} H=\left[\begin{array}{ccc} g\left(w_{1}x_{1}+b_{1}\right) & \cdots & g\left(w_{N}x_{1}+b_{N}\right)\\ \vdots & \ddots & \vdots \\ g\left(w_{1}x_{M}+b_{1}\right) & \cdots & g\left(w_{N}x_{M}+b_{N}\right) \end{array}\right], \end{array}$$

where *β* is the matrix of middle-to-output weights and *T* is the target matrix.

In (8), weights *w*_*i*_ and bias *b*_*i*_ are assigned random float number and *g*(·) is selected as sigmoid function; therefore the output of middle perceptron can be determined very fast, which is *H* in (7).

The remaining work is minimum square error estimation:

(9) $$\begin{array}{c} \underset{\beta }{\min}\left\|\;H\beta -T\;\right\|. \end{array}$$

The smallest norm least squares solution for (9) can be calculated by applying the definition of the Moore-Penrose generalized inverse; the solution is as follows:

(10) $$\begin{array}{c} \hat{\beta }=H^{-1}T, \end{array}$$

where *H* ^−1^ is the generalized inverse of matrix *H*.

The least squares solution of (10) based on Kuhn-Tucker conditions can be written as follows:

(11) $$\begin{array}{c} \beta =H^{T}{\left(\;\frac{1}{C}+HH^{T}\;\right)}^{-1}T, \end{array}$$

where *H* is the middle layer output, *C* is regulation coefficient, and *T* is the expected output matrix of instances.

Therefore, the output function is

(12) $$\begin{array}{c} f\left(\;x\;\right)=h\left(\;x\;\right)H^{T}{\left(\;\frac{1}{C}+HH^{T}\;\right)}^{-1}T. \end{array}$$

The kernel matrix of ELM can be defined as follows:

(13) $$\begin{array}{c} M=HH^{T}:m_{ij}=h\left(\;x_{i}\;\right)h\left(\;x_{j}\;\right)=k\left(\;x_{i},x_{j}\;\right). \end{array}$$

Therefore, the output function *f*(*x*) of kernel extreme learning machine can be expressed as follows:

(14) $$\begin{array}{c} f\left(\;x\;\right)=\left[\;k\left(\;x,x_{1}\;\right),\ldots ,k\left(\;x,x_{N}\;\right)\;\right]{\left(\;\frac{1}{C}+M\;\right)}^{-1}T, \end{array}$$

where *M* = *HH* ^*T*^ and *k*(*x*, *y*) is the kernel function of perceptrons in middle layer.

We adopt three kernel functions in this paper; they are as follows:

 POLY: for some positive integer *d*,
  (15) $$\begin{array}{c} k\left(\;x,y\;\right)={\left(\;1+\langle \;x,y\;\rangle \;\right)}^{d}. \end{array}$$
 RBF: for some positive number *a*,
  (16) $$\begin{array}{c} k\left(\;x,y\;\right)=\exp\left(\;-\frac{\langle \left(x-y\right),\left(x-y\right)\rangle }{\left(2a2\right)}\;\right). \end{array}$$
 MLP: for a positive number *p* and negative number *q*,
  (17) $$\begin{array}{c} k\left(\;x,y\;\right)=\tanh\left(\;p\langle \;x,y\;\rangle +q\;\right). \end{array}$$

Compared with ELM, KELM performs similarly to or better than ELM, and KELM is more stable [25]. Compared with SVM, KELM spends much less time without performance loses.

### 3.3. Bagging Technique

By applying ensemble learning [26] to our approach, classifier can obtain better classification performance when dealing with overfitting problem brought by small training set. We apply bagging technique to enhance KELM classifier. Bagging technique seeks to promote diversity among the methods it combines. In the initialization procedure, we adopt multiple different kernel functions and different parameters. Therefore, a group of classifiers can be built for bagging technique implementation.

When applying a group of KELMs with bagging method, each KELM is trained independently and then those KELMs are aggregated via a majority voting technique. Given a training set TR = {(*x*_*i*_, *y*_*i*_)∣*i* = 1,2,…, *n*}, where *x*_*i*_ is extracted features from retinal images and *y*_*i*_ is corresponding diagnosis result, we then build *M* training datasets randomly to construct *M* KELMs bagging independently.

The bootstrap technique is as follows:

 init:
   given training datasets TR
   
*M* distinctive KELM_*i*_, *i* = 1,2, 3,…, *M*
 training:
   construct subtraining datasets {STR_*i*_∣*i* = 1,2,…, *M*} form TR with resampling randomly and replacement
   train KELM_*i*_ with STR_*i*_
 classification:
   calculate hypothesis *H*_*i*_ of ELM_*i*_, *i* = 1,2, 3,…, *M*
   perform majority voting of *H*_*i*_

## 4. Experiments and Results

### 4.1. Messidor Database and Evaluation Criteria

For empirical experiment, we use public Messidor dataset [27] that consists of 1151 instances. Images are of 45-degree field of view and three different resolutions (440*∗*960, 2240*∗*1488, and 2304*∗*1536).

Each image is labeled 0 or 1 (negative or positive diagnostic result). 540 images are labeled 0; the remnants are labeled 1. Many researches did 5-fold (or 10-fold) cross-validation. Thus, 80% of database is training dataset and the remaining 20% instances are testing dataset. We train Classification and Regression Tree (CART), radial basis function (RBF) SVM, Multilayer Perceptron (MLP) SVM, Linear (Lin) SVM, and *K* Nearest Neighbor (KNN) with 80% of database and the remaining 20% is as testing dataset.

For the proposed active learning (AL) classifier, we use 10%–20% of database as initial training dataset and give it 10%–15% of database as queries made by committee. Therefore, 20%–35% of database is used to train active learning classifier in total, and the remaining 65%–80% of database is testing dataset. We also train ELM and KELM with 20%–35% of database, and the remaining 65%–80% of database is testing dataset. Therefore, ELM, KELM, and our approach are trained with the same amount of labeled instances; the results can prove the availability of kernel technique, bagging technique, and active learning. Committee contains all classifiers which were mentioned in this paper.

In short, we use 80% of Messidor database to train 5 classifiers, and we cut more than half the training instances to validate ELM, KELM, and our approach. Details are presented in Section 4.3. Each classifier was tested 10 times. The recommendations of the British Diabetic Association (BDA) are 80% sensitivity and 95% specificity. Therefore, accuracy, sensitivity, and specificity are compared among those classifiers.

Sensitivity, accuracy, and specificity are defined as follows:

(18) Sensitivity=TPTP+FN,Accuracy=TP+TNTP+FP+TN+FN,Specificity=TNFP+TN,

where TP, FP, TN, and FN are the true and false positive and true and false negative classifications of a classifier.

### 4.2. Retinal Image Features

Table 2 lists every feature extracted from retinal images with feature information. The retinal images are mapped in a 19-dimensional space.

The details of image features used in Messidor database are listed as follows:

1. *Quality Assessment*. Messidor database contains sufficient quality image for a reliable diagnosis result. After detecting vessel system, the box count values can be calculated for a supervised learning classifier. The vessel segmentation algorithm is based on Hidden Markov Random Fields (HMRF) [28].
2. *Prescreening*. Images are classified as abnormal or to be needed for further processing. Every image is split into disjoint subregions and inhomogeneity measure [29] is extracted for each subregion. Then a classifier learns from these features and classifies the images.
3. *MA Detection*. Microaneurysms appear as small red dots and they are hard to find efficiently. The MA detection method used in Messidor database is based on preprocessing method and candidate extractor ensembles [30].
4. *Exudate*. Exudates are bright small dots with irregular shape. By following the likely complex methodology as for microaneurysm detection [30], it combines preprocessing methods and candidate extractors for exudate detection [31].
5. *Macula Detection*. Macula is located in the center of the retina. By extracting the largest object from image with brighter surroundings [32], the macula can be detected effectively.
6. *Optic Disc Detection*. Optic disc is anatomical structure with circular shape. Ensemble-based system of Qureshi et al. [33] is used for optic disc detection.
7. *AM/FM-Based Classification*. The Amplitude-Modulation Frequency-Modulation method [34] decomposes the green channel of the image and then signal processing techniques are applied to obtain representations which reflect the texture, geometry, and intensity of the structures.

### 4.3. Experiment Results

CART, RBF, MLP, Lin, KNN, AL, KELM, and ELM are compared in experiments. For CART, RBF, MLP, Lin, and KNN, 80% of labeled retinal images are offered to these 5 classifiers as training dataset. The parameters of those 5 classifiers are determined by grid search on training dataset. To AL, KELM and ELM are also used as grid-search method to set hidden layer. The MATLAB R2015a version is used in this paper.

**Figure 5:**
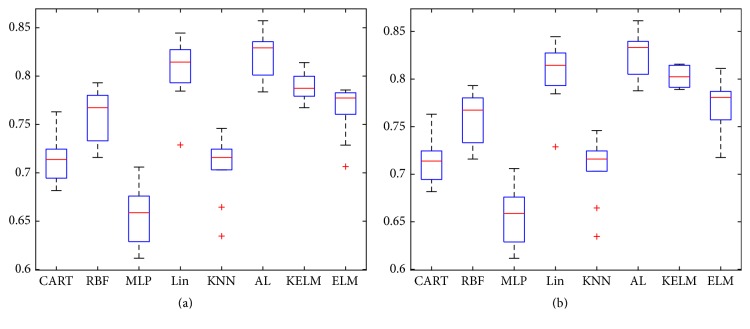
(a) Using 10% of Messidor dataset as initial training dataset and the committee proposes 10% of dataset as queries and (b) using 10% of Messidor dataset as initial training dataset and the committee proposes 15% of dataset as queries.

Figure 5 shows the boxplot of normalized correct classifications. In Figure 5(a), AL has 115 instances (10% of Messidor database) for initial training and 115 more instances (10% of Messidor database) are queries from committee. Therefore, 230 (20% of Messidor database) instances are used in training AL in total. For testing kernel, bagging technique, and active learning, KELM and ELM are offered 230 training instances (20% of Messidor database). Other 5 classifiers are trained with 920 instances (80% of Messidor database).

KELM is classified more accurately than ELM by the kernel technique. Bagging technique and active learning method further elevate classification accuracy of KELM. Comparing AL with other 7 classifiers, its correct classification is about 2%~20% higher than other classifiers in Figure 5(a), and the training dataset of AL is only 25% of other 5 classifiers. MLP, CART, and KNN are the worst three classifiers. RBF performs a little better than ELM, but RBF has three times more labeled instances than ELM. Lin performs better than ELM and KELM, but it is slightly lower than AL.

Similarly, in Figure 5(b), active learning and other classifiers have been tested again. KELM gives more correct classification results and AL is better than both ELM and KELM. Comparing AL with other classifiers, AL achieves better classification accuracy and AL only needs 287 labeled instances for training.

**Figure 6 fig6:**
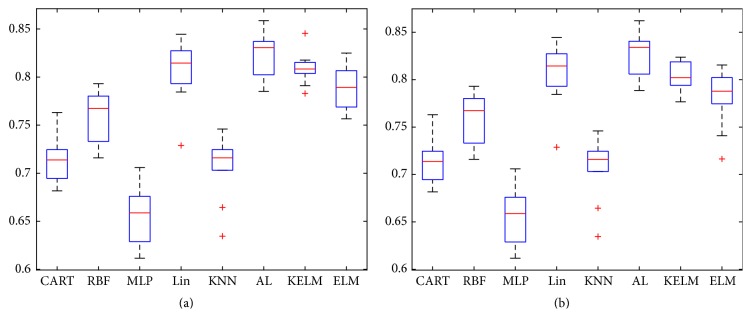
(a) Using 15% of Messidor dataset as initial training dataset and the committee proposes 10% of dataset as queries and (b) using 15% of Messidor dataset as initial training dataset and the committee proposes 15% of dataset as queries.

In Figure 6, CART, RBF, MLP, Lin, and KNN are exactly the same as in Figure 5. In Figure 6(a), a bigger initial training dataset (15%) is used to train AL and 25% labeled instances are given to KELM and ELM as training dataset. In Figure 6(b), 30% labeled instances are given to KELM and ELM for training. In Figure 6, kernel technique helps ELM to produce more correct classification results, and the active learning method still further boosts KELM. In Figure 6, Lin performs closely to AL, but Lin has nearly triple the size of training dataset. Therefore, the disadvantage of Lin is the need of massive manual work.

**Figure 7:**
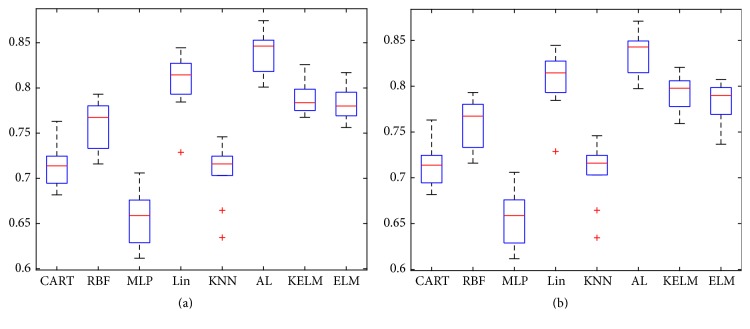
(a) Using 20% of Messidor dataset as initial training dataset and the committee proposes 10% of dataset as queries and (b) using 20% of Messidor dataset as initial training dataset and the committee proposes 15% of dataset as queries.

Figure 7 shows 20% of labeled instances as initial training dataset for AL. Figure 7 proves the same conclusion as shown in Figures 5-6; kernel technique, bagging technique, and active learning are effective and efficient for improving ELM. It should be noticed that KELM and ELM are tested twice which Figures 5(b) and 6(a) present. Similarly, Figures 6(b) and 7(a) also present twice the comparison results of ELM and KELM.  Table 4 lists results of Figures 5 [Fig fig6]–7 in detail.

Table 3 contains 5 columns; the names of classifiers are attached with experiment parameters. For instance, KNN_80 is that KNN classifier is trained with 80% of instances. The max, min, and mean are calculated from 10 runs. AL_10_15 is that 10% of labeled instances are as initial training dataset and 15% of labeled instances are queries form committee.

In Table 3, the lower limit and mean value of AL_10_10 are the highest in column. The upper limit of AL_20_10 is the highest in column.

In Table 4, mean values of sensitivity and specificity are listed for all classifiers. The first column of Table 4 is corresponding experiment as Table 3. Second column is mean values of sensitivity, and third column is mean values of specificity. All mean values are statistical result of 10 runs. Sensitivity mean values are between 0.74 and 0.82; specificity mean values are between 0.83 and 0.92.

### 4.4. Discussions

In this section, we present two issues about experiment results: (1) what are the advantages of KELM? (2) Is the proposed method suitable for medical implement?

The KELM is ELM with kernel technique; this approach is similar to SVM. The kernel technique can map original data (linear inseparable) into a new space (higher dimensional space but linear separable) for a linear classifier. The major contribution of KELM is that kernel technique helps ELM to face a high dimensional classification problem which is faster than kernel-SVM when solving the same problem. Especially in this paper, the Messidor dataset contains 18 features; all classifiers must give a hypothesis in 18-dimensional space.

The proposed method is suitable for implementation. The recommendations of the British Diabetic Association (BDA) [35] are 80% sensitivity and 95% specificity. The test results of our method are close to those two standards.

## 5. Conclusion

In this paper, an active learning classifier is presented for further reducing diabetic retinopathy screening system cost. Classic researches did 5- or 10-fold cross-validation which implies that massive diagnosis results should be prepared beforehand. Unlike other state-of-the-art methods, we focus on further reducing cost. We use kernel extreme learning machine to deal with classification problem in high dimensional space. For solving overfitting problem brought by small training set, we adapt ensemble learning method. By using active learning with QBC, the ensemble-KELM learns from manual diagnosis result by necessary queries.

Our approach and other comparative classifiers had been validated on public diabetic retinopathy dataset. Kernel technique and bagging technique are also tested and analyzed. Empirical experiment shows that our approach can classify unlabeled retinal images with higher accuracies than other comparative classifiers, but the size of training dataset is much smaller than other comparative classifiers. With the consideration of implementation, the performance of our approach is close to the recommendations of the British Diabetic Association.

## Figures and Tables

**Table 1:** Essential detection of DR screening.

Detection target	Detail information
Microaneurysm	An extremely small aneurysm, it looks as tiny red dots in retinal image.

Exudates	Fat or lipid leak from aneurysms or blood vessels, it looks as small and bright spots with irregular shape.

Inhomogeneity	Regions of retina are different and unusual.

Macula	The macula is an oval-shaped pigmented area near the center of the retina of the human eye.

Optic disc	The optic disc is the point of exit for ganglion cell axons leaving the eye.

**Table 2:** Image features of Messidor dataset [18].

Feature	Feature information
(0)	The binary result of quality assessment. 0: bad quality; 1: sufficient quality.

(1)	The binary result of prescreening, where 1 indicates severe retinal abnormality and 0 its lack.

(2–7)	The results of MA detection. Each feature value stands for the number of MAs found at the confidence levels alpha = 0.5 ⋯ 1, respectively.

(8–15)	Contain the same information as (2–7) for exudates. However, as exudates are represented by a set of points rather than the number of pixels constructing the lesions, these features are normalized by dividing the number of lesions with the diameter of the ROI to compensate different image sizes.

(16)	The Euclidean distance of the center of the macula and the center of the optic disc to provide important information regarding the patient's condition. This feature is also normalized with the diameter of the ROI.

(17)	The diameter of the optic disc.

(18)	The binary result of the AM/FM-based classification.

(19)	Class label. 1: containing signs of DR (accumulative label for the Messidor classes 1, 2, and 3); 0: no signs of DR.

**Table 3:** Details of Figures 5 [Fig fig6]–7 (accuracy).

	Classifiers	Max	Min	Mean
Figures 5 [Fig fig6]–7	CART_80	0.775	0.693	0.725
	RBF_80	0.805	0.728	0.770
	MLP_80	0.718	0.623	0.669
	Lin_80	0.856	0.741	0.818
	KNN_80	0.758	0.646	0.720

Figure 5(a)	AL_10_10	0.872	0.799	0.838
	KELM_20	0.832	0.785	0.808
	ELM_20	0.804	0.725	0.783

Figure 5(b)	AL_10_15	0.880	0.807	0.846
	KELM_25	0.803	0.776	0.790
	ELM_25	0.799	0.705	0.761

Figure 6(a)	AL_15_10	**0.886**	0.804	0.836
	KELM_25	0.834	0.771	0.798
	ELM_25	0.813	0.745	0.778

Figure 6(b)	AL_15_15	0.871	0.797	**0.851**
	KELM_30	0.812	0.765	0.792
	ELM_30	0.804	0.705	0.769

Figure 7(a)	AL_20_10	0.874	0.800	0.839
	KELM_30	0.833	0.774	0.795
	ELM_30	0.824	0.763	0.790

Figure 7(b)	AL_20_15	0.878	**0.812**	0.843
	KELM_35	0.837	0.776	0.811
	ELM_35	0.823	0.753	0.800

Max of column		**0.886**	**0.812**	**0.851**

**Table 4:** Sensitivity and specificity.

Classifiers	Sensitivity mean	Specificity mean
CART_80	77.64%	83.10%
RBF_80	78.07%	86.17%
MLP_80	74.41%	84.52%
Lin_80	80.29%	88.96%
KNN_80	77.13%	88.13%
AL_10_10	81.69%	91.46%
KELM_20	79.44%	90.81%
ELM_20	78.92%	90.03%
AL_10_15	82.38%	91.54%
KELM_25	78.43%	90.72%
ELM_25	77.80%	90.26%
AL_15_10	82.67%	92.11%
KELM_25	80.54%	91.23%
ELM_25	79.87%	90.78%
AL_15_15	82.63%	92.08%
KELM_30	81.88%	91.91%
ELM_30	81.35%	90.35%
AL_20_10	82.78%	91.58%
KELM_30	81.83%	90.03%
ELM_30	81.10%	89.61%
AL_20_15	82.63%	91.72%
KELM_35	81.95%	90.18%
ELM_35	81.21%	88.96%

## References

[1] Kollias A. N., Ulbig M. W. (2010). Diabetic retinopathy: early diagnosis and effective treatment. *Deutsches Arzteblatt International*.

[2] Venkatnarayan K., Pboyle J., Jthompson T. (2003). Lifetime risk for diabetes mellitus in the United States. *Journal of the American Medical Association*.

[3] Memon M., Memon S., Bakhtnizamani N. (2014). Sight threatening diabetic retinopathy in type—2 diabetes mellitus. *Pakistan Journal of Ophthalmology*.

[4] Driver V. R., Fabbi M., Lavery L. A., Gibbons G. (2010). The costs of diabetic foot: the economic case for the limb salvage team. *Journal of Vascular Surgery*.

[5] Li R., Sshrestha S., Dlipman R. (2014). Diabetes self-management education and training among privately insured persons with newly diagnosed diabetes—United States, 2011-2012. *Morbidity and Mortality Weekly Report*.

[6] Chan W. C., Lim L. T., Quinn M. J., Knox F. A., McCance D., Best R. M. (2004). Management and outcome of sight-threatening diabetic retinopathy in pregnancy. *Eye*.

[7] Cuadros J., Bresnick G. (2009). EyePACS: an adaptable telemedicine system for diabetic retinopathy screening. *Journal of Diabetes Science and Technology*.

[8] Gardner G. G., Keating D., Williamson T. H., Elliott A. T. (1996). Automatic detection of diabetic retinopathy using an artificial neural network: a screening tool. *British Journal of Ophthalmology*.

[9] Hscanlon P., Pwilkinson C., Jaldington S. (2009). *Screening for Diabetic Retinopathy*.

[10] Sinthanayothin C., Kongbunkiat V., Phoojaruenchanachai S. Automated screening system for diabetic retinopathy.

[11] Gardner G. G., Keating D., Williamson T. H., Elliott A. T. (1996). Automatic detection of diabetic retinopathy using an artificial neural network: a screening tool. *The British Journal of Ophthalmology*.

[12] Liew G., Egan C. A., Rudnicka A. (2014). Evaluation of automated software grading of diabetic retinopathy and comparison with manual image grading—an accuracy and cost effectiveness study. *Investigative Ophthalmology & Visual Science*.

[13] Canny J. (1986). A computational approach to edge detection. *IEEE Transactions on Pattern Analysis and Machine Intelligence*.

[14] Hyvarinen A. (1999). Fast and robust fixed-point algorithms for independent component analysis. *IEEE Transactions on Neural Networks*.

[15] Alberti K. G. M. M., Zimmet P. Z. (1998). Definition, diagnosis and classification of diabetes mellitus and its complications. Part 1: diagnosis and classification of diabetes mellitus. Provisional report of a WHO consultation. *Diabetic Medicine*.

[16] Vijan S., Hofer T. P., Hayward R. A. (2000). Cost-utility Analysis of screening intervals for diabetic retinopathy in patients with type 2 diabetes mellitus. *Journal of the American Medical Association*.

[17] Shotliff K., Duncan G. (2006). Diabetic retinopathy: summary of grading and management criteria. *Practical Diabetes International*.

[18] http://archive.ics.uci.edu/ml/.

[19] Antal B., Hajdu A. (2014). An ensemble-based system for automatic screening of diabetic retinopathy. *Knowledge-Based Systems*.

[20] Dabramoff M., Mreinhardt J., Rrussell S. (2010). Automated early detection of diabetic retinopathy. *Ophthalmology*.

[21] Fleming A. D., Goatman K. A., Philip S., Olson J. A., Sharp P. F. (2007). Automatic detection of retinal anatomy to assist diabetic retinopathy screening. *Physics in Medicine and Biology*.

[22] Sopharak A., Uyyanonvara B., Barman S. (2009). Automatic exudate detection for diabetic retinopathy screening. *ScienceAsia*.

[23] Krogh A., Vedelsby J. (1995). Neural network ensembles, cross validation, and active learning. *Neural Information Processing Systems*.

[24] Sseung H., Opper M., Sompolinsky H. Query by committee.

[25] Huang G.-B., Zhu Q.-Y., Siew C.-K. (2006). Extreme learning machine: theory and applications. *Neurocomputing*.

[26] Huang G.-B., Zhou H., Ding X., Zhang R. (2012). Extreme learning machine for regression and multiclass classification. *IEEE Transactions on Systems, Man, and Cybernetics, Part B: Cybernetics*.

[27] Dietterich T. G. (2000). An experimental comparison of three methods for constructing ensembles of decision trees: bagging, boosting, and randomization. *Machine Learning*.

[28] Kovács G., Hajdu A. Extraction of vascular system in retina images using averaged one-dependence estimators and orientation estimation in hidden markov random fields.

[29] Antal B., Hajdu A., Maros-Szabó Z., Török Z., Csutak A., Peto T. (2012). A two-phase decision support framework for the automatic screening of digital fundus images. *Journal of Computational Science*.

[30] Antal B., Lázár I., Hajdu A. (2012). An ensemble approach to improve microaneurysm candidate extraction. *Signal Processing and Multimedia Applications*.

[31] Nagy B., Harangi B., Antal B., Hajdu A. Ensemble-based exudate detection in color fundus images.

[32] Antal B., Hajdu A. (2011). A stochastic approach to improve macula detection in retinal images. *Acta Cybernetica*.

[33] Qureshi R. J., Kovacs L., Harangi B., Nagy B., Peto T., Hajdu A. (2012). Combining algorithms for automatic detection of optic disc and macula in fundus images. *Computer Vision and Image Understanding*.

[34] Agurto C., Murray V., Barriga E. (2010). Multiscale AM-FM methods for diabetic retinopathy lesion detection. *IEEE Transactions on Medical Imaging*.

[35] Leese G. P. (1997). Retinal photography screening for diabetic eye disease.

